# On the search of the ideal barrier membrane for guided bone regeneration

**DOI:** 10.4317/jced.54767

**Published:** 2018-05-01

**Authors:** Jordi Caballé-Serrano, Antonio Munar-Frau, Octavi Ortiz-Puigpelat, David Soto-Penaloza, Miguel Peñarrocha, Federico Hernández-Alfaro

**Affiliations:** 1 Department of Oral and Maxillofacial Surgery, School of Dentistry, Universitat Internacional de Catalunya, Barcelona, Spain; 2 Department of Oral Surgery, School of Medicine and Dentistry, University of Valencia, Spain

## Abstract

**Background:**

GBRs are essential procedures in implant dentistry and periodontology where barrier membranes play an important role by isolating soft tissue and allowing bone to grow. Not all membranes function the same way, as they differ from their origin and structure, it is important to understand how membranes behave and differ one from others in order to achieve a predictable treatment.

**Material and Methods:**

A systematic search on Medline by two independent reviewers was performed for articles published until July 2017 reporting the characteristics or properties of barrier membranes. The question that preceded the search was designed according to PICO rules.

**Results:**

A total of 124 articles were initially identified from electronic searching. After abstract/full-text review, 21 were included for a systematic review. According to the extracted data and article analysis, barrier membranes should fulfill the following criteria in order to success: biocompatibility, space maintaining, occlusive function, easy - handling and a bioactivation friendly property. With the development of new biomaterials and surfaces, a great advance in this area is expected.

**Conclusions:**

It has been clearly described that biocompatibility is the most important requirement to take into account when choosing a membrane, but other factors such as space maintaining capacity, cell oclusiveness, easy handling and bioactivation friendly materials are the ones that will fulfill our necessities.

** Key words:**Barrier membrane, guided bone regeneration, dental implantology, oral surgery, collagen membrane, biomaterial.

## Introduction

Guided bone regeneration (GBR) and guided tissue regeneration (GTR) are nowadays essential procedures in implant dentistry and periodontology. Their main objective is to restore the lost tissues creating an ideal condition to place an implant or maintain a tooth. For a successful bone regeneration, the bone defect needs to be isolated from the soft tissues permitting bone to grow, taking a minimum of 4 - 6 weeks for periodontal tissues and 16 – 24 weeks for bone (1,2). From the first GTR procedures described in the 1950s to nowadays, a need to find the ideal biomaterial for each case has existed; from a small periodontal regeneration where simple resorbable membrane are used, to vast defects where a titanium mesh should be placed (1,3,4). Today, the use of a resorbable membrane is extended in the clinical practice compared to the non-resorbable membranes such as expanded polytetrafluorethylene membrane (ePTFE) (5,6).

Although sometimes non-resorbable membranes are the choice of election, resorbable membrane are used in most cases due to their main advantages; similar results to non – resorbable materials, decreased morbidity, less risk of membrane exposure, no additional costs and no need of a second surgery (7). Even though resorbable membranes do not need a second surgery, they suffer from a low tensile strength which can be a limitation when compared to ePTFE membranes or a titanium mesh, lowering the ability of space maintenance (8). According to the degradation ability of membranes, newly chemically cross – linked collagen membranes have shown to present lower degradation rates. Nevertheless, having a longer resorption time does not guarantee greater bone regeneration compared to natural collagen (9).

It is important to mention that depending on the tissue origin and processing technique, the membrane will present a different degradation time and a different structure. These physical characteristics might alter the response of the evolving tissues (10,11). When performing regeneration procedures we must take into account few aspects of the barrier membrane such as biocompatibility, ability to create space, cell occlusiveness, tissue integration and handling as well as the resorption time. In other words, a membrane should be stiff and biocompatible enough to avoid the soft tissue penetration or collapse into the regeneration area (4,8). Numerous membranes are appearing in the market pursuing the concept of an ideal membrane that could cope with all types of regeneration. Some examples are the lately developed PLGA membranes (2,12) or silk based membranes (8) to new 3D (12) PLGA CAD/CAM printed materials.

The aim of this review is to reveal the ideal properties of a barrier membrane in terms of biocompatibility, occlusive properties, dimensional properties “space maintainers”, handling bioactivation properties and to show what tendencies are to come in the field of membranes in bone regeneration.

## Material and Methods

-Development of a protocol

A bibliographic search protocol was developed before commencing the review. This protocol included a definition of the question, a search strategy, inclusion criteria, a determination of the outcome measures, screening methods, and data analysis.

-Defining the focused question

The following issue was defined: “which main criteria should a barrier membrane fulfill”?.

-Search strategy

Using the Medline PubMed database, the articles were searched including publications up to July 2017. The combinations of different terminologies were included ( Table 1).

**Table 1:**
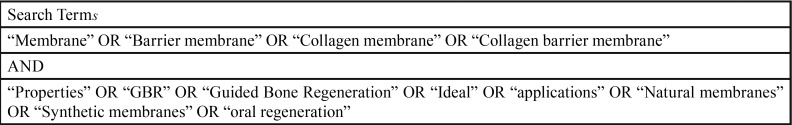
Search of free text terms used for the electronic search in Medline-Pubmed.

-Criteria for study selection and inclusion

The study selection included articles published in English, describing *in vitro* studies, clinical trials and reviews. All studies including the guided bone regeneration (GBR) concept, excluding guided tissue regeneration. All types of membranes were included in the search.

-Outcome measure determination

The aim of this review was to assess the main criteria that a barrier membrane should fulfill, therefore looking for an ideal barrier membrane for bone regeneration.

-Screening method

Two independent reviewers (JCS and AMF) chose titles and abstracts independently. The selection was based on: “which main criteria should a barrier membrane fulfill”?. After answering this question the full text articles were obtained. Disparity regarding the inclusion criteria was resolved by a meeting between the authors.

-Data extraction and analysis

**Figure 1:**
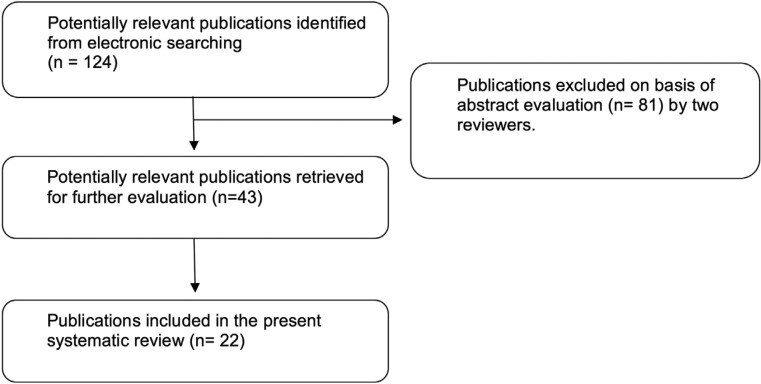
Flow chart of the screened relevant publications.

Data extraction and analysis was performed as shown in  Table 1 and in Figure 1. Briefly, keywords concerning barrier membranes were selected and once having the full text of selected articles and having read them, 22 papers were selected.

## Results

Barrier membranes are crucial in new bone formation. When aiming to regenerate, a resorbable or a non – resorbable membrane should be used depending on the technique and defect area (13). There has not been described the ideal membrane yet. Authors differ in their opinions; nevertheless, an ideal membrane should maintain its barrier function enough time for new bone formation, and if possible should be resorbable, so a second surgery would not be needed, thus reducing the morbidity.

Non - resorbable membranes do not suffer from a degradation process when placed in the body, but require a second surgery in order to remove them. Although e-PTFE has been considered the gold standard membrane for GBR and GTR due to its stability and biological resistance, the inconvenient of a second surgery, and high membrane exposure rate, induced resorbable membranes to appear. On the other hand they do not suffer from a degradation process, making them one of the main membranes we must compare to (13).

In 1992 Scantlebury described five main criteria that membrane should fulfill which are: biocompatibility, the ability to create space, cell occlusiviness, tissue integration and easy – handeling (4). Therefore, an ideal bone regeneration membrane should be synthetic, biocompatible, easy to handle and resorbable (2). The morphological structure, biological stability and the ability to activate grow factors are also key factors we must take into account to gain a major bone volume.

The five main criteria that a membrane should fulfill are the following:

1. Biocompatible: the interaction between the membrane and the tissues must affect positively the surrounding tissues, leading to the healing of the defect. If the membrane is resorbable, should either degrade or integrate into the host tissues, decreasing the incompatibility that a cross – linking membrane can cause (2,12,14-16).

2. Space maintainer: a membrane must be stable enough and create space to facilitate bone formation (12).

3. Occlusive: to prevent the ingrowth of soft tissues into the regeneration site but at the same time allow oxygen, fluids and bioactive substances for cell growth to reach the defect (16).

4. Easy – handling: a membrane should not be too stiff because it would not integrate with the tissue or could create dehiscence of the soft tissues; or too malleable making it difficult to work with (12).

5. Bioactivation friendly: this feature of membranes is nowadays not into consideration. However, new strategies for bone regeneration are being developed which bring the membranes into the next level, not only having a passive role but an active role into the regeneration site (2).

**Figure 2:**
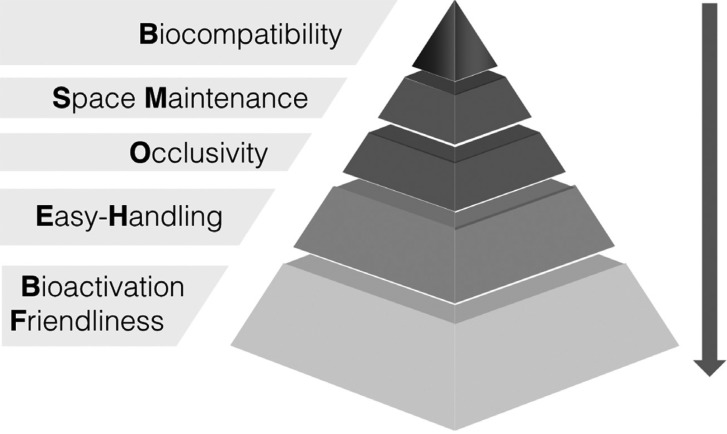
Pyramid with the 5 main criteria that barrier membranes should fulfill.

Graphically, these results can be displayed in a pyramid as shown in Figure 2. Publications included in the review are summarized in  Table 2, Table 2 continue.

**Table 2:**
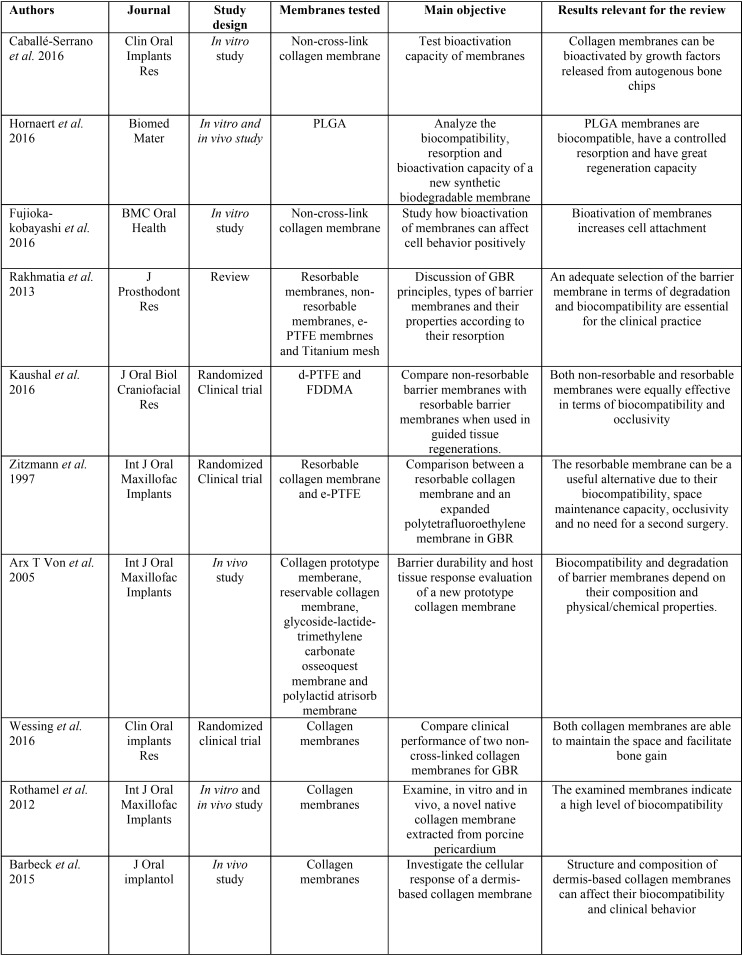
Summary of data extracted from publications included in the review. Authors, journal of publication, study design, membranes tested, main objective and results relevant for the review are included.

**Table 2 continue:**
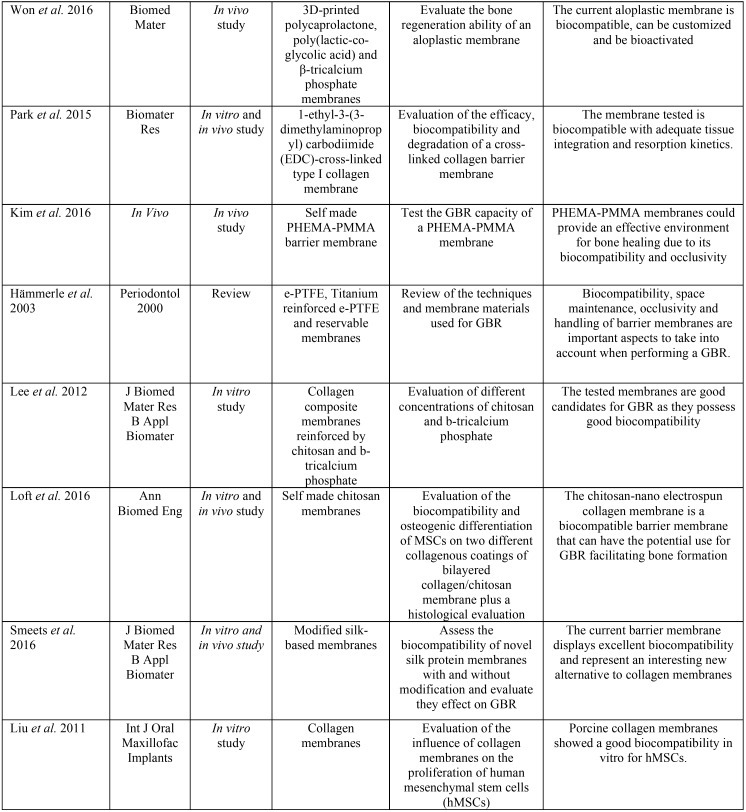
Summary of data extracted from publications included in the review. Authors, journal of publication, study design, membranes tested, main objective and results relevant for the review are included.

## Discussion

The aim of this review was to analyze the main criteria that a barrier membrane should fulfill, to establish the ideal properties of barrier membrane as well as to analyze the tendencies when talking about GBR, on the search of the ideal membrane. Non – resorbable membranes have been widely used for decades due to their ability of long term space maintenance, from the treatment of critical size osseous defects to socket grafting, demonstrating that GBR can lead to a successful regeneration (13).

When comparing the degradation properties, according to the companies given information, porcine natural collagen membranes are the fastest to resorb (4 – 8 weeks), whereas cross-linked membranes and bone lamina membranes offer more margins in terms of resorption (4 – 6 months and 5 – 8 months respectively) (2). Apart from the surgical technique used, and as previously said, the properties of a membrane are crucial to reach the needed regeneration. As described by Scantlebury in 1992 a barrier membrane must fulfill five main criteria: biocompatibility, the ability to create space, cell occlusiviness, tissue integration and easy – handeling (4).

Lately PLGA membranes have been appearing. Biodegradable synthetic barrier PLGA membranes consist in a 2 layers membrane; a thin dense film to prevent the invasion of soft connective tissue cells, and a thick micro – fibrous layer that induces the stabilization of the blood clot allowing bony cells to colonize the membrane. PLGA membrane might be a safer and more predictable alternative for GBR due to its biocompatibility and abilities to differentiate soft tissues and maintaining its barrier function for an estimated time of 16 weeks. PLGA membranes turn to be stable as they maintain their weight for 12 weeks before beginning to lose it. Its pH remains stable for 12 weeks *in vitro*. When implanted in rats, after 26 weeks almost any part of a membrane could be seen, so it seems to be a good correlation between the *in vitro* and *in vivo* study. According to the inflammation cells less macrophages and multi - nucleated giant cells appeared in contact with the membrane, indicating its biocompatibility and use for GBR (2).

In the last years there has been a willing to improve the existing membranes or prefabricated membranes in order to achieve a better biocompatibility, and a greater capacity to form new bone, which should be one of the main standards when choosing a membrane (15). When pre – coating membranes to potentiate the activity of cells (1,3), a collagen membrane would be ideal to pre – coat due to their ability to adsorb the TGF – ß. Moreover, collagen membranes are a safer option in dehiscence defects making them nowadays suitable for almost any regeneration procedure (9). It is also possible to produce modified membranes using chitosan, collagen or beta-tricalcium phosphate improving some of the properties of membranes. Chitosan coated with collagen nanofibers is useful as a natural biocomposite polymer for GBR purposes and has the capacity to accelerate bone formation, considered with the biocompatibility one of the major objectives in GBR (17,18).

From now on, researchers are looking for other materials that can allow surface modification, such as silk membranes modified by calcium phosphate. In this case no inflammatory changes were shown and new bone formation advancing from the periphery could be detected. Due to its high biocompatibility, silk – based membranes offer an interesting alternative (19). According to Sang – woon Lee in its 2014 study, silk used in the oral cavity if well prepared by an acid treatment can be used as a barrier membrane for GBR, being its action the same as a collagen membrane, showing a small inflammatory reaction and new bone formation, being a good candidate as a drug carrier. Therefore, the proper development of this material is essential due to its properties and low price (8).

In the last years with the introduction of the CAD/CAM systems and complemented with CBCT scans, there has been a willing to develop 3D printed membranes. In the study of J – Y won 2016, they used a 3D – printed PCL/PLGA/ B- TCP membrane which showed comparable results to collagen membranes; therefore this new 3D – printed system might become an alternative to other membranes when either a GBR or GTR is required (12).

One thing we must consider when choosing a membrane is its morphological structure and processing. As Rothamel et al. described in their article, when comparing RPCM (Remotis Pericardium Collagen Membrane) to Bio – Gide, both membranes showed a comparable tissue integration (10). Although cross – linking increases the degradation time, it might compromise the biocompatibility of the membrane due to the crosslinking agents which produce an inflammatory response leading in some cases to a failure of the tissue integration (14). In general, collagen membranes show good results, nevertheless there is a trend to show that cross-linked membranes are less biocompatible. In Qin Liu’s study, authors showed that porcine collagen membranes had a great *in vitro* biocompatibility and a moderate to low cytotoxicity. Proliferation rates were adequate, but if required, a pre – washing of the membrane could reach higher proliferation rate (20).

As any material, we must take into account their properties and ideal use scenario, but we must never forget that when talking about surgery we should be as less invasive as possible, therefore it is important not to raise big flaps or second surgery flaps for membrane removal, lowering patients morbidity (21). This review has limitations. To perform a review to asses the state of the art of a specific topic implicates the systematic search of literature to be as evidence based as possible. Nevertheless, the present review could not be performed as a systematic review using all PRISMA guidelines due to the singularity of the present report.

## Conclusions

From the first development of barrier membranes until today there has been a great development in membrane science. Although nowadays natural collagen membranes are the ones that offer the wider range indications, we must consider that they are no suitable for every procedure, and that the clinician should be aware of the situation required to choose the right membrane.

It has been clearly described that biocompatibility is the most important requirement to take into account when choosing a membrane, but other factors such as space maintaining capacity, cell oclusiveness, easy handling and bioactivation friendly materials are the ones that will fulfill our necessities.

Future studies are needed to clarify how pre – coated membranes function, to analyze new materials and new methods on making the ideal membrane for a patient. It is therefore important not to forget the main criteria that a membrane should fulfill.
